# Supplementary material for: The Antioxidant and Anti-Inflammatory Effects of Flavonoids from Propolis via Nrf2 and NF-κB Pathways
Source: Foods. 2022 Aug 13;11(16):2439. doi: 10.3390/foods11162439 (PMC9407528; doi:10.3390/foods11162439)
Supplement: Supplementary file 1 [file foods-11-02439-s001.zip › foods-1734436-supplementary.pdf]

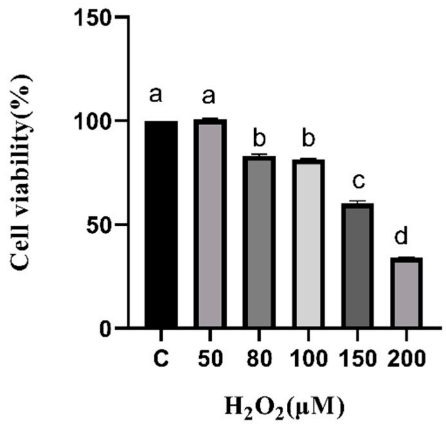

Figure S1. Cell viability of CCK-8 assays based on H9c2 cells. H9c2 cells were stimulated with different concentrations of H_2O_2 (0, 50, 80, 100, 150, 200 μM) for 1 h and then the cell viability was detected by CCK-8 assay. Values are expressed as the mean ± SEM (n = 6). Values with different letters in the different column demonstrated significant difference at $p < 0.05$.

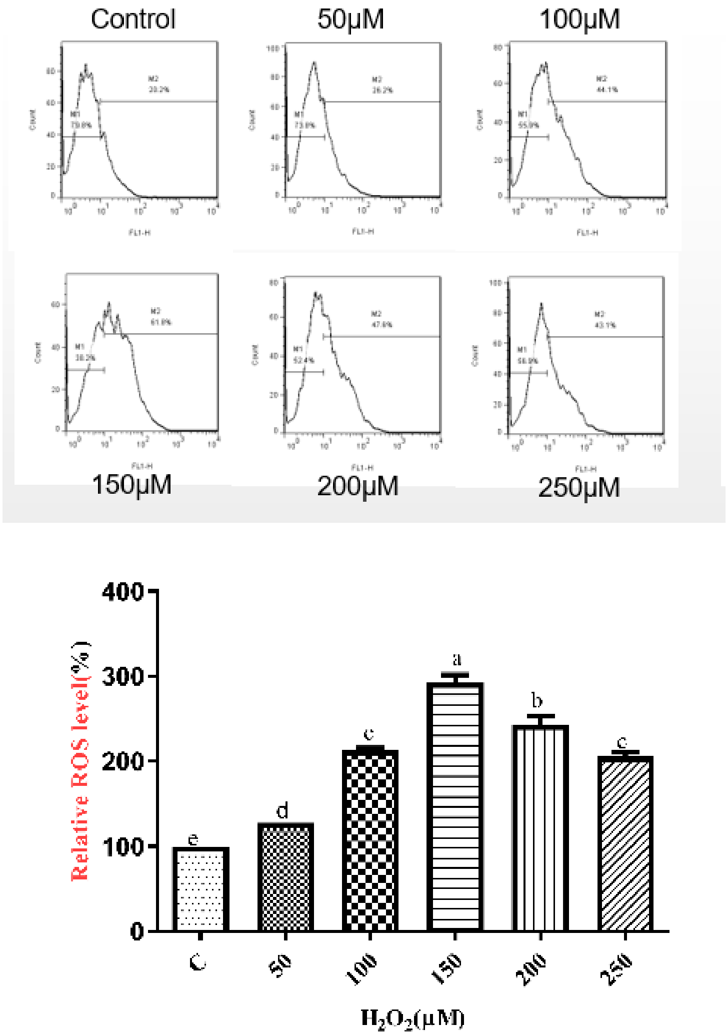

Figure S2. ROS level based on H_2O_2-induced H9c2 cells. H9c2 cells were stimulated with different concentrations of H_2O_2 (0, 50, 100, 150, 200, 250 μM) for 1 h and then the ROS level was detected by Flow Cytometry. Values are expressed as the mean ± SEM (n = 3). Values with different letters in the different column demonstrated significant difference at $p < 0.05$.

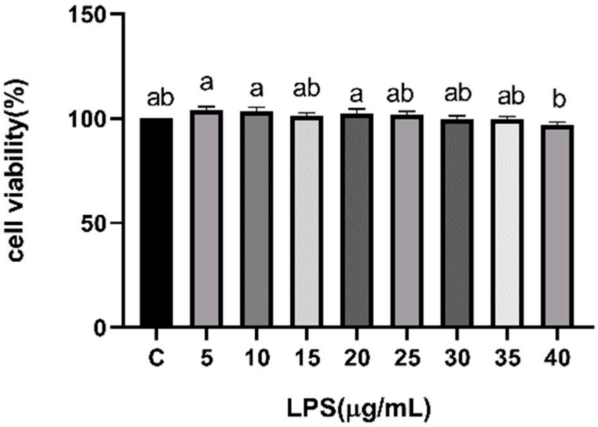

Figure S3. Cell viability of CCK-8 assays based on H9c2 cells. H9c2 cells were stimulated with different concentrations of LPS (0, 5, 10, 15, 20, 25, 30, 35, 40 μg/mL) for 12 h and then the cell viability was detected by CCK-8 assay. Values are expressed as the mean ± SEM (n = 6). Values with different letters in the different column demonstrated significant difference at $p < 0.05$.

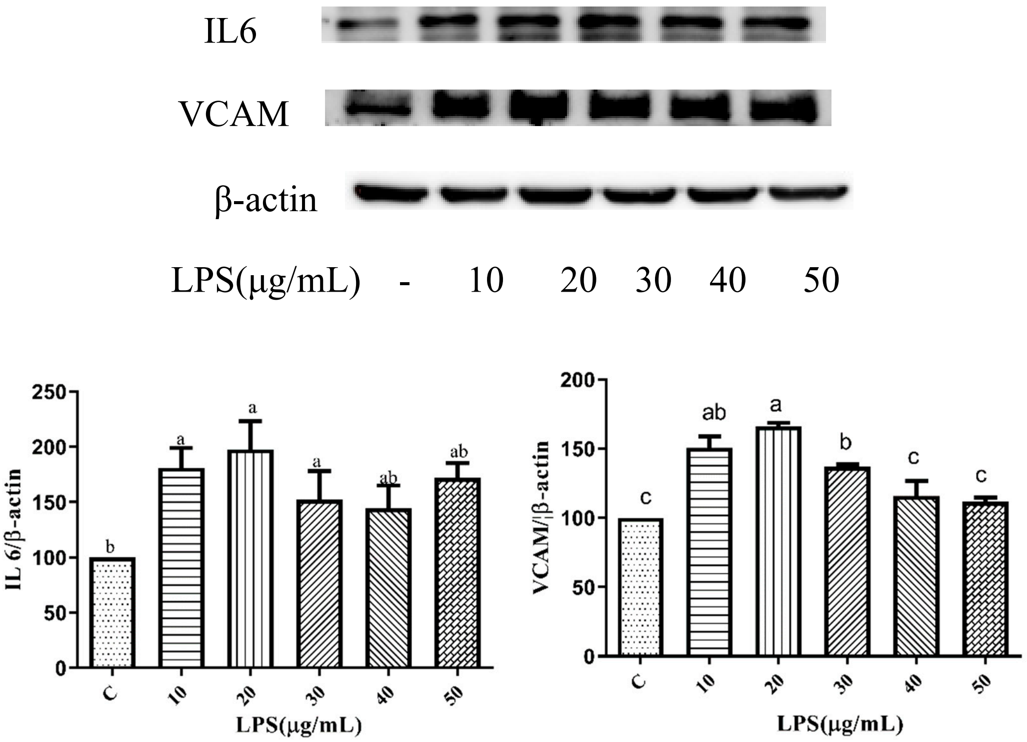

Figure S4. The expression of pro-inflammatory protein (VCAM1 and IL-6) based on LPS-induced H9c2 cells. Values are expressed as the mean ± SEM (n = 3). Values with different letters in the different column demonstrated significant difference at $p < 0.05$.

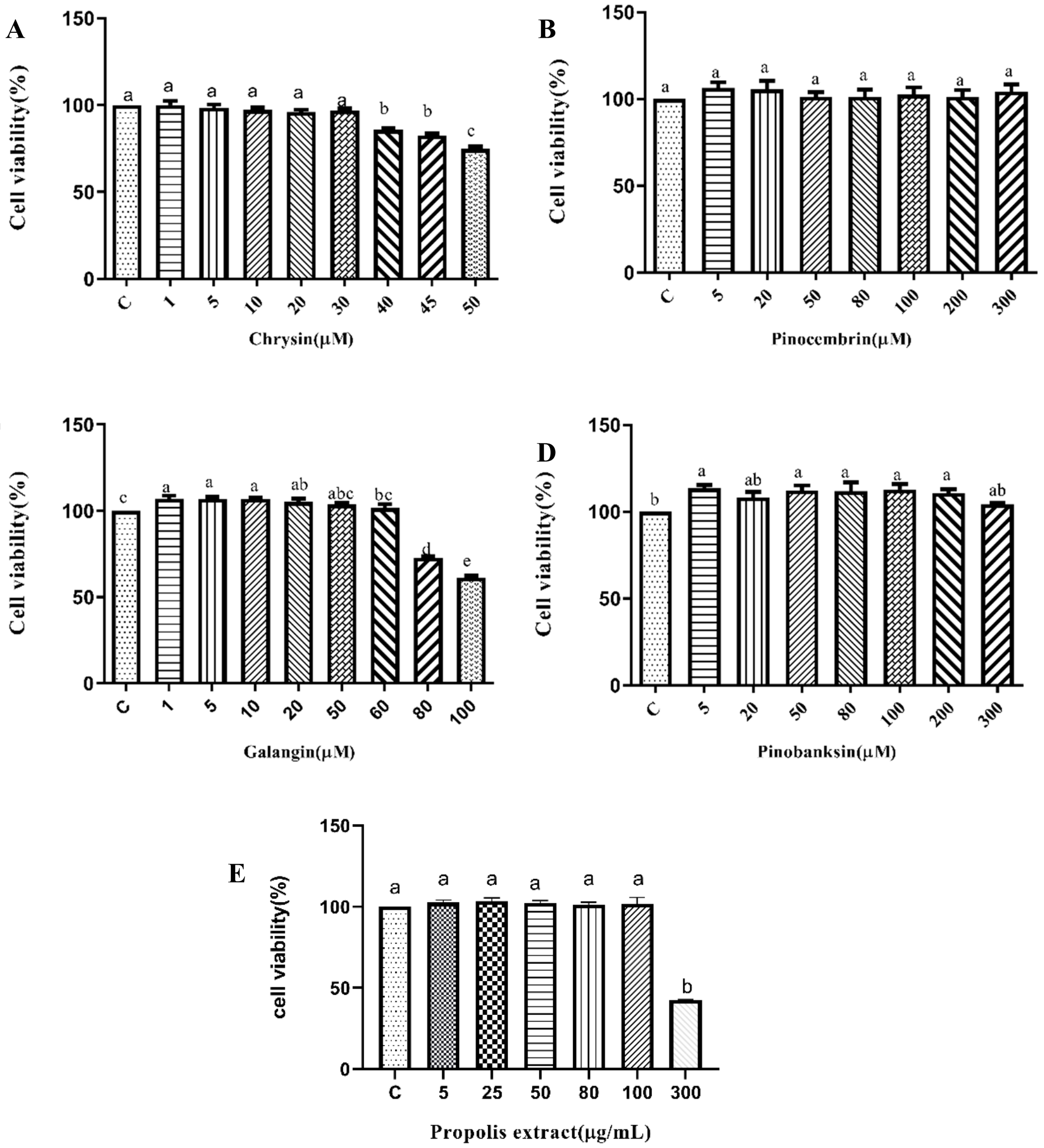

Figure S5. Cell viability of CCK-8 assays based on H9c2 cells. H9c2 cells were stimulated with different concentrations of individual flavonoids (chrysin, pinocembrin, galangin and pinobanksin) and propolis extract for 12 h and then the cell viability was detected by CCK-8 assay. (A) Cell viability of chrysin-induced H9c2 cells. (B) Cell viability of pinocembrin-induced H9c2 cells. (C) Cell viability of galangin-induced H9c2 cells. (D) Cell viability of pinobanksin -induced H9c2 cells. (E) Cell viability of propolis extract-induced H9c2 cells. Values are expressed as the mean ± SEM (n = 6). Values with different letters in the different column demonstrated significant difference at $p < 0.05$.
